# Generative Models for Global Collaboration Relationships

**DOI:** 10.1038/s41598-017-10951-5

**Published:** 2017-09-11

**Authors:** Ertugrul Necdet Ciftcioglu, Ram Ramanathan, Prithwish Basu

**Affiliations:** 1 U.S. Army Research Lab, Adelphi, MD 20783 USA; 2 Raytheon BBN Technologies, Cambridge, MA 02138 USA

## Abstract

When individuals interact with each other and meaningfully contribute toward a common goal, it results in a *collaboration*. The *artifacts* resulting from collaborations are best captured using a hypergraph model, whereas the *relation* of *who has collaborated with whom* is best captured via an *abstract simplicial complex* (SC). We propose a generative algorithm GENESCs for SCs modeling fundamental collaboration relations. The proposed network growth process favors attachment that is preferential not to an individual’s *degree*, i.e., how many people has he/she collaborated with, but to his/her *facet degree*, i.e., how many maximal groups or *facets* has he/she collaborated within. Based on our observation that several real-world facet size distributions have significant deviation from power law–mainly since larger facets tend to *subsume* smaller ones–we adopt a data-driven approach. We prove that the facet degree distribution yielded by GENESCs is power law distributed for large SCs and show that it is in agreement with real world co-authorship data. Finally, based on our intuition of collaboration formation in domains such as collaborative scientific experiments and movie production, we propose two variants of GENESCs based on *clamped* and *hybrid* preferential attachment schemes, and show that they perform well in these domains.

## Introduction

Many large endeavors in society such as scientific discoveries and production of motion pictures are a result of collaboration. Typically, individuals collaborate to form teams, for example, a scientific paper is written jointly by a team of researchers. Also, smaller teams can collaborate to form larger groups. Examples of the latter include a movie production house containing teams of artists, directors, and crew; a disaster relief mission requiring interactions between teams of medical rescue workers, fire-fighters, and law enforcement officials with some common agents serving as gateways; and a major scientific discovery happening with the coming together of research over a series of papers, which typically have some common authors. The main goal of this paper is to understand the fundamental characteristics of the underlying global collaboration structures that exist in collaborative fields such as scientific research and movie production.

In modeling collaboration structures, the basic collaborative unit could either be the *relation* underlying the collaboration or the *output* or *artifact* from the collaboration (e.g. paper or movie). The difference in the resulting structure is best illustrated with a simple example. Suppose authors *a*, *b*, *c*, and *d* write three papers with authorships (a,b,c), (a,b), (c,d). Then a structure based on the collaboration artifact is identical to the set of papers, whereas one based on the collaboration relation is (a,b,c), (c,d). In other words, the collaboration relation structure ignores (a,b) since (a,b,c) already captures the fact that any subset of it, in particular (a,b), has collaborated. Previous studies of collaboration networks have overwhelmingly focused on the artifact-based structure^1–9^. The relation-based structure, is instead able to capture the *social* aspects of collaboration, which is interesting in its own right.

*Hypergraphs* (HG) are suitable for expressing “richer than pairwise” relationships between collaborators^2, 6^; however, we believe they best model the *artifacts* of the collaboration–each by a hyperedge. The collaboration *relation* on the other hand is closed under the subset operation. A perfect match for succinctly capturing such a property is the *abstract simplicial complexes* (SC), which in simplest terms is a collection of sets closed under the subset operation.

The primary distinction between HGs and SCs is that in the case of the latter, a “simplex” of dimension *k* (modeling a (*k* + 1)-ary collaboration relation) *subsumes* all subset simplices of dimension *k* − 1, and so on recursively. Consequently, if an HG is used to model a collaboration *relation*, the distributions of sizes and degrees turn out to be non-trivially skewed compared to the relation structure itself, or equivalently, the SC representation thereof.

In this paper, we propose a new generative algorithm GENESCs for SCs that models the fundamental relations underlying large-scale collaboration. GENESCs is primarily based on preferential attachment–not with an individual’s *degree* (how many people has he/she collaborated with) but rather with his/her *facet degree* (how many maximal groups or *facets* has he/she collaborated within). Unlike graphs, where a node’s degree can capture its first order local connectivity properties, in SCs, there are two key metrics to consider–a node’s facet degree and a facet’s size. Based upon our observation that several real-world *facet size* distributions have significant deviation from power law–predominantly due to the fact that larger facets tend to *subsume* smaller ones–we adopt a data-driven approach. We “seed” GENESCs with a facet size distribution (from input data) and grow the SC randomly facet-by-facet to generate a final SC with a *facet degree distribution* that matches real data.

Note that we sample the facet size distribution as input instead of the artifact (hyperedge) size distribution since we want to generate the underlying SC, and not the Hypergraph, and there may be several discordant facet size distributions resulting from a single hyperedge size distribution based on how the collaboration relation is structured.

Our key contributions are summarized below:

1. Systematic characterization of the nature of subsumption in real world global collaboration networks. (Introduction Section and Supplementary Information)
2. An efficient generative algorithm GENESCs to generate realistic SCs with matching facet degree distributions, given only their facet size distributions. (Methods Section.)
3. An analytic proof that the facet degree distribution of generated SCs is power law distributed, matching empirical studies, with an exponent $\alpha =2+\frac{1}{c\,s-1}$, where *c* is the average facet density (ratio of the number of facets to the number of nodes) and *s* is the average facet size. *cs* is the average facet degree, and thus the average number of collaborations in which a node participates. (Results Section, Theorem 0.1)
4. Validation using empirical statistical analysis that the generated SCs have low Kolmogorov-Smirnov and Total-Variation distances from the real data. (Results Section)
5. Adaptations of the core facet-based preferential attachment kernel in GENESCs to model some observed variations in real world collaboration structures. (Methods Section)

Interestingly, we demonstrate (and give analytical justification for the fact) that when GENESCs generates facets one after another with their sizes randomly drawn from the facet size distribution of the target real data set given as input, the probability of occurrence of subsumptions during this random growth process is negligible. This does not contradict our observation that subsumption phenomena is common in real collaboration artifact data. In reality, subsumptions occur over sequentially added hyperedges, whereas in GENESCs, we already start with the pre-subsumed facet-based representation, hence further distortion is not required. This feature of GENESCs is a valuable benefit by virtue of using facets as opposed to hyperedges in the sampling process.

## Modeling Global Collaboration Relationships

Standard *graphs* are insufficient to capture group phenomena since they only model *binary* relations between individuals. A generalization of graphs, namely, the *hypergraph* has been proposed to address this shortcoming^2, 6^. A hypergraph *H* = (*G*, *E*) comprises a set of nodes *V* and *hyper-edges* $E\subseteq {2}^{V}$ to model higher order (or *super-binary*) relations.

Insights about the structure of a large collaboration network can be drawn by examining its “artifacts”, i.e., papers, movies, etc., and the underlying distributions of *hyperedge size* (the number of nodes belonging to a hyperedge, e.g., number of co-authors in a paper) and *hyper-degree* (the hyper-degree of a node is the number of hyperedges it belongs to, e.g., number of movies an actor has acted in).

We believe that equally interesting insights can emerge from an understanding of the collaboration relation which focuses on the social aspects of the collaboration, namely the set of collaborating individuals. Such a question can be answered by examining the underlying higher-order global collaboration structure, which is not concerned about the specific products of the collaboration. For example, if *a*, *b*, and *c* have collaborated as a group (*a*, *b*, *c*), then sparser collaboration relationships (*a*, *b*) or (*b*, *c*), even if they occurred, do not add much value if our goal is to understand the number of maximal groups that a person has collaborated in.

Such information is indeed buried in the “collaboration artifact network”, i.e., the hypergraph, but typically, statistical properties of the higher-order global collaboration structure cannot be trivially determined from those of the hypergraph. In the worst case, the representation complexity of hypergraphs grows exponentially, since *k* collaborating individuals can build as many as 2^*k*^ − 1 different artifacts. Since we are only interested in the fact that these *k* individuals collaborated on at least one project, the artifact network may be too unwieldy for analysis.

### Abstract Simplicial Complexes

The basic structure of the underlying collaboration can be modeled by an *abstract simplicial complex* (*SC*). A set-system *SC* of non-empty finite subsets of a universal set *S* is an abstract simplicial complex if for every set $X\in SC$, and every non-empty subset $Y\subset X$, $Y\in SC$–thus the set-system is “closed” under subset operation^10^. Therefore, a simplicial complex captures the basic nature of the collaboration, i.e., who all have worked together on common tasks, instead of the artifacts that have been produced by such a collaboration, i.e., papers or movies.

Consider the example in Supplemental Information Fig. 1–suppose *a*, *b*, *c*, and *d* are four authors who have co-authored five papers among them including single-author papers–this co-authorship is denoted by a hypergraph consisting of five hyperedges: *H* = {(*a*, *b*), (*a*, *b*, *c*), (*b*, *c*), (*a*, *d*), (*a*)}. The collaboration structure would be represented by simplicial complex with facets {(*a*, *b*, *c*), (*a*, *d*)} which *subsumes* the other three simplexes because if *a*, *b*, and *c* collaborate with each other, all subsets of them do so as well. While *a* and *c* have not explicitly collaborated *separately*, they have collaborated with each other in presence of *b* in the paper denoted by (*a*, *b*, *c*). Essentially, a simplicial complex consists of a set of maximal simplexes or *facets*.

**Figure 1:**
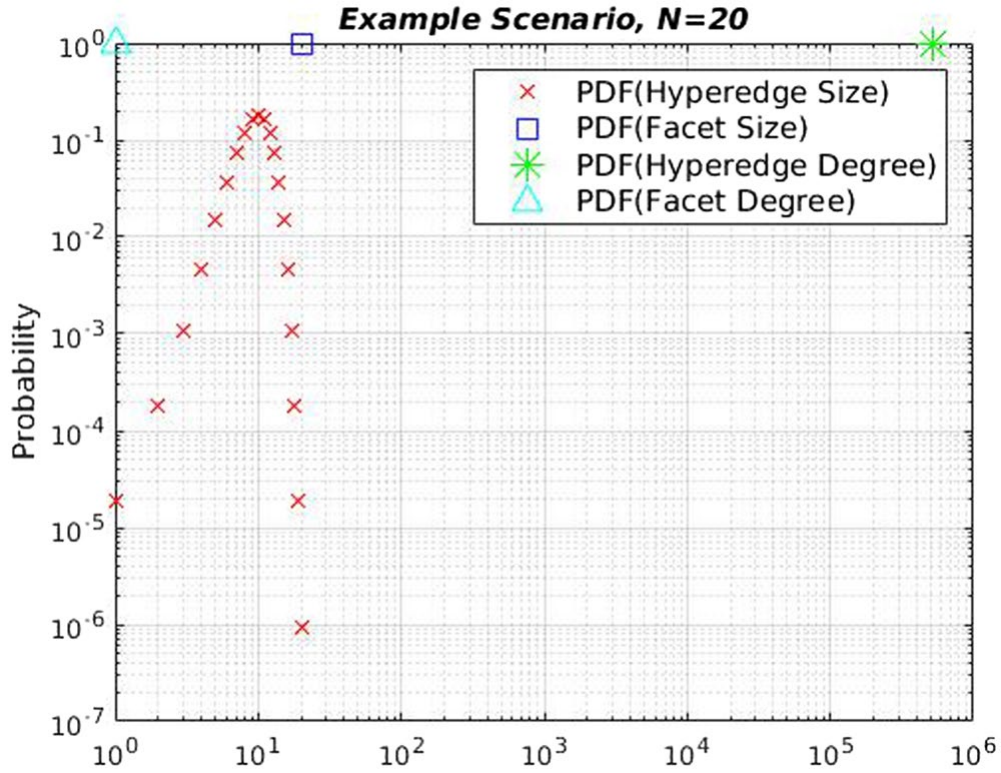
A hypothetical case with extreme subsumptions.

In previous work, we have shown how simplicial complexes can be effectively used to model collaboration networks^11–13^. For a collaborative group denoted by a facet, two basic metrics are *facet size* (how many people belong to that collaboration) and a node’s *facet degree* (how many maximal collaborations or facets does that node belong to).

## Modeling Subsumptions

The basic difference between hypergraphs and simplicial complexes can be explained by the phenomenon of *subsumptions*. In the previous example, hyperedges (*a*, *b*) and (*b*, *c*) get *subsumed* by the largest hyperedge (*a*, *b*, *c*), which is a *facet* in SC. Similarly, facet (*a*, *d*) subsumes (*a*).

In theory, subsumptions can be very pronounced. Consider a large research project with *N* participating faculty members. Consider the situation where each faculty member has one single-author paper, one paper written with every other faculty member, a paper with each set of two other distinct faculty members, and so on. Finally, assume that all of these *N* authors collaborate to write a joint paper together. Clearly, there are 2^*N*^ − 1 distinct hyperedges in total–*N* single author papers, $$(\begin{array}{c}N\\ 2\end{array})$$ two-author papers, and $$(\begin{array}{c}N\\ k\end{array})$$ *k*-author papers, in general. Since each node has exactly 2^*N*−1^ hyperedges, the hyperedge degree distribution is given by the impulse function ${\delta }_{{d}_{i}{\mathrm{,2}}^{N-1}}$, where *δ*_*i*,*j*_ denotes the Kronecker delta function, and *d*_*i*_ is the degree of researcher *i*. On the other hand, hyperedge size distribution is a non-monotonic function which is proportional to $\frac{N!}{(N-k)!k!}$, centered around $\frac{N}{2}$. In contrast, in the simplicial complex representation, the largest collaboration is the only *facet*, so all nodes have facet degree 1, hence the facet degree distribution is ${\delta }_{{d}_{i}\mathrm{,1}}$.

Moreover, the only facet has size *N*, hence the facet size distribution is ${\delta }_{{s}_{j},N}$, where *s*_*j*_ is number of authors in paper *j* (See Fig. 1). There is significant discrepancy between the two distributions due to the intense degree of subsumption–since all faculty members collaborate with each other on one paper, the other smaller collaborations are directly implied by the former. Note that the deviation would be larger if there were multiple papers with exactly the same authors, since the hyperedge count would increase without affecting facet statistics.

The above example demonstrated a case where many different hypergraph instances associated with *N* nodes may map to only one simplicial complex, i.e. a many-to-one mapping between the set of different hypergraphs to one simplicial complex representation. Yet, one may think that once a specific hypergraph is given, the corresponding simplicial complex can be simply obtained from it by following the subset closure operation. However, in reality, while working with datasets, one typically expects only the distributional statistics to be given. We next demonstrate that starting from a given hyperedge size distribution one might end up with multiple simplicial complex representations with drastically different facet size distributions even for a fixed number of nodes. In fact, the total variation distance *D*_*TV*_ (a commonly used distance metric to compare two different distributions, i.e., normalized statistical distance between two distributions which measures sum of differences over the support set, formally defined in the Results Section) among the various feasible non-isomorphic simplicial complexes might approach 1, which is the maximum value that can be defined between two distributions, as *N* → ∞.

**Figure 2:**
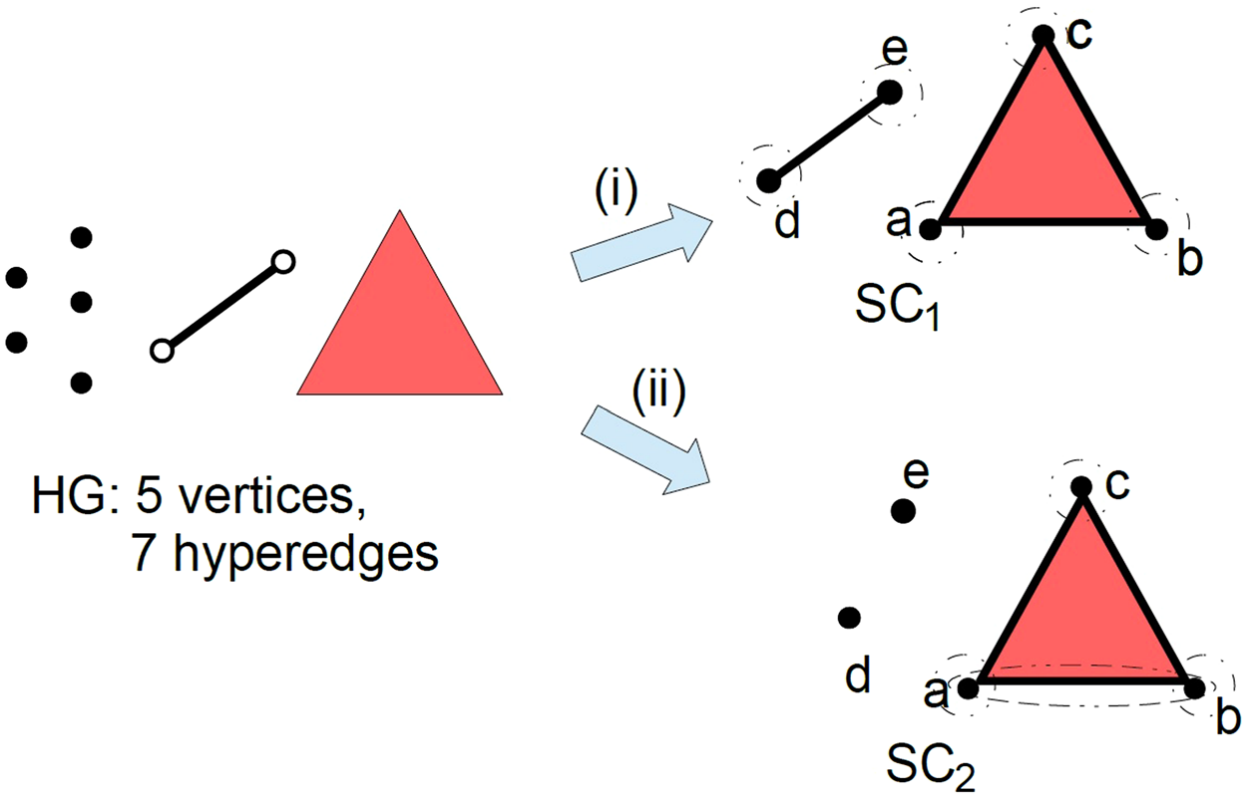
Two of the possible different Simplicial Complexes and the corresponding facet size *distributions z*(*s*), *resulting from a given Hyperedge size distribution f*_*h*_(*s*) ≈ (0.714,0.143,0.143) with *N* = 5 nodes: (i) *SC*_1_ = {(*a*, *b*, *c*), (*d*, *e*)}, ${z}_{S{C}_{1}}(s)=\mathrm{(0,}\,\mathrm{0.5,}\,\mathrm{0.5)}$ (ii) *SC*_2_ = {(*a*, *b*, *c*), (*d*), (*e*)}, ${z}_{S{C}_{2}}(s)=\mathrm{(0.}\bar{6},0,0.\bar{3})$. Total variation distance (*D*_*TV*_) between the two facet size distributions: $0.\bar{6}$ The dashed contours depict hyperedges which were subsumed in the facet representations.

Consider a given hyperedge size distribution *f*_*h*_(.). Let us denote the maximum hyperedge size in *f*_*h*_(.) by *H*, and assume that *N* = 2*H* − 1, where again *N* = |*V*| denotes the number of nodes. Next, consider a hypergraph consisting of a total of 2*H* + 1 hyperedges, with 2*H* − 1 hyperedges of size one, and one hyperedge each of size *H* − 1 and *H*. That is, ${f}_{h}\mathrm{(1)}=\frac{2H-1}{2H+1},{f}_{h}(H-\mathrm{1)}=\frac{1}{2H+1}$ and ${f}_{h}(H)=\frac{1}{2H+1}$. A small scale illustration of this scenario with *N* = 5 and *H* = 3 is given in Fig. 2. Given this distribution, it may be possible that the two large hyperedges are disjoint and do not possess any nodes in common, hence in the SC representation there is one facet with size *H* − 1 and one facet with size *H*, together spanning all *N* = 2*H* − 1 of the nodes. Accordingly, all the smaller (single node) hyperedges are subsumed by the two larger facets since every node belongs to a larger collaboration (e.g. *SC*_1_ in Fig. 2). On the other hand, it could also be the case that the larger hyperedge of size *H* subsumes the one of size *H* − 1. Then, *H* − 1 of the 2*H* − 1 hyperedges of size one do not belong to the larger facets, and hence are disjoint. Overall, there are *H* − 1 size one facets and one size *H* facet in the simplicial complex representation. (e.g. *SC*_2_ in Fig. 2).

It can be observed from Fig. 2 that the facet size distribution of *SC*_1_ is an *H*-dimensional vector (0, …, 0.5, 0.5) with non-zero entries at *H* − 1 and *H*; and the facet size distribution of *SC*_2_ is $(\frac{H-1}{H},\mathrm{0,}\ldots ,\frac{1}{H})$ with ${f}_{S}(H)=\frac{1}{H}$. The total variations (please see the Results Section for a formal definition) between two distributions are $0.5(\frac{H-1}{H}+0.5+|0.5-\frac{1}{H}|)$, which converges to 1 as *N* (and hence *H*) grows. Even if the condition *N* = 2*H* − 1, i.e., *H* ≈ *N*/2 may be found to be restricting in the sense that *H* may not grow as much, alternative examples and expressions can be constructed.s

For example, with *N* = 3*H* − 2, where the maximum hyperedge is of size *H* ≈ *N*/3, assume we have a hypergraph on *N* nodes, which consists of one hyperedge of size *H*, two hyperedges of size *H* − 1, and 3*H* − 2 hyperedges of size one. This corresponds to the hyperedge size distribution ${f}_{h}\mathrm{(1)}=\frac{3H-2}{3H+1},{f}_{h}(H-\mathrm{1)}=\frac{2}{3H+1}$ and ${f}_{h}(H)=\frac{1}{3H+1}$. Then, if all large facets are disjoint they cover all nodes and one has a facet size distribution of $\mathrm{(0},0,\mathrm{....0.}\bar{6},0.\bar{3})$, whereas if the two facets of size *H* − 1 differ by only one node and are both subsumed by the one of size *H*; 2*H* − 2 of the singleton nodes remain disjoint, and the facet size distribution for this scenario is $(\frac{2H-2}{2H-1},\,\mathrm{0,}\,\mathrm{...},\frac{1}{2H-1})$ and the total variation would still approach 1 for large enough *H*.

The above examples clearly demonstrate that if one in interested in understanding global collaboration relationship structures represented as simplicial complexes, starting from hyperedge size statistics to generate SCs may result in wildly discordant structures. Hence, in our generative algorithm GENESCs (in the Methods Section 1) we take the facet size distribution as input, instead.

We note that we consider an evolving model of collaborations where a “facet” emerges (i.e., at time *t* it is indeed a facet in the Simplicial Complex) and remains a facet until it is subsumed at a later time *t*′ > *t* by a bigger facet modeling the occurrence of a collaboration involving a *superset* of the participants. Thus at time *t*′, in the resulting ultimate simplicial complex structure, the (older) subsumed facet does not exist anymore, as it merely becomes a “face” of the larger facet. Similarly, if at time *t*′ a collaboration occurs that involves a *subset* of participants of an older collaboration that had occurred at time *t* < *t*′, then the new collaboration is just a face of the older facet and is instantly subsumed by the latter. To that end, until the end of the growth process, we term every facet in the complex a *transient facet*, in the sense that it has a probability of getting subsumed. On the other hand, at the end of the growth process, any surviving facet will be a (permanent) facet.

**Figure 3:**
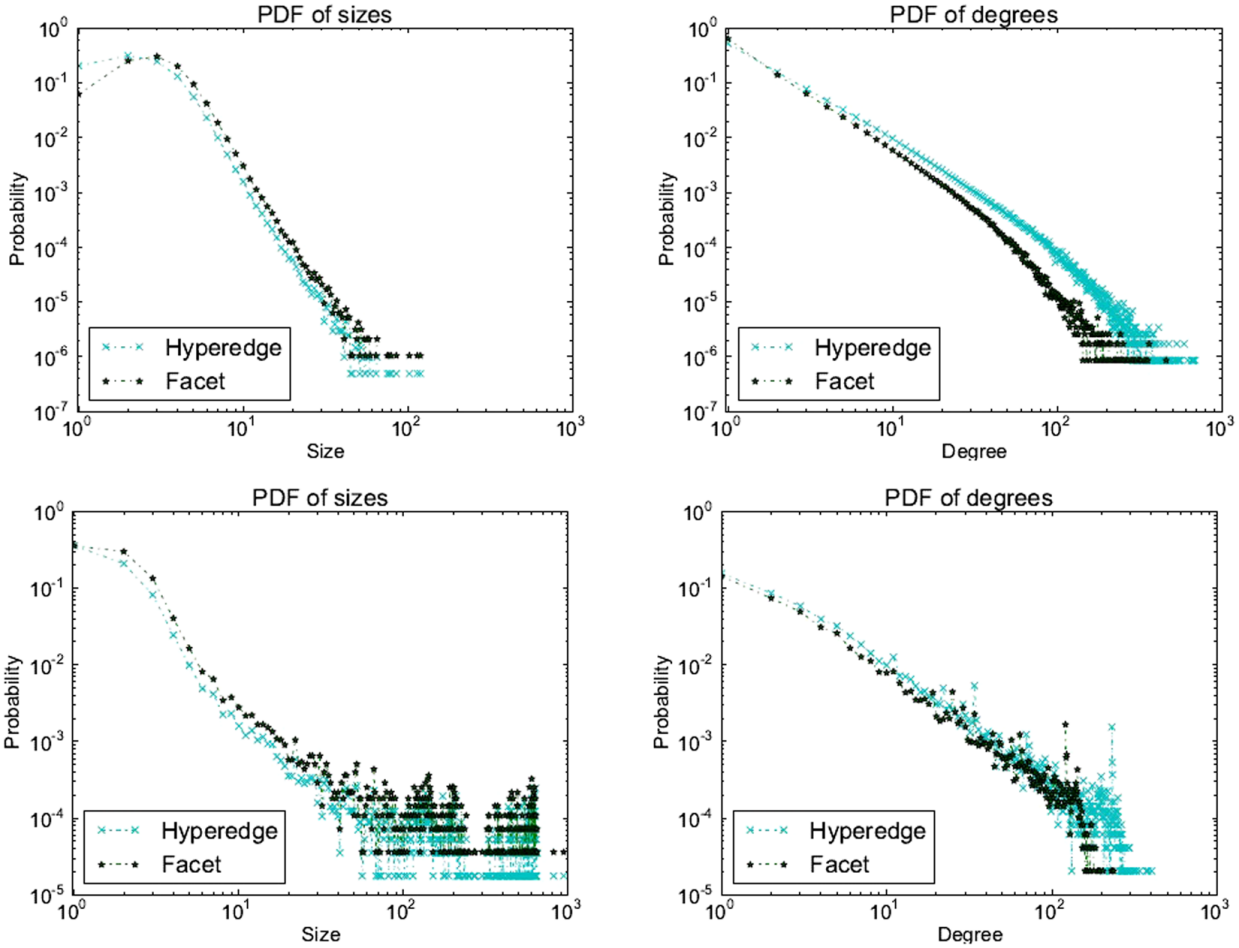
Hypergraph vs. Simplicial Complex metrics for (i) DBLP(top pair): (**a**) size and (**b**) degree; and (ii) Physical Review D (bottom pair): (**c**) size and (**d**) degree.

While the above examples can be regarded as rather unlikely events, subsumption does occur quite frequently in real world collaboration. Figures 3a,b illustrate statistics for both hypergraph and SC models of DBLP co-authorship data. While the tail of the facet size distribution obeys a *power law*, the head, where significant probability mass is concentrated, does not. In particular, a significant number of singleton authors get subsumed by the larger collaborations they participate in. Additionally, the slopes of the facet degree and hyperedge degree distributions are different. This can also be attributed to *subsumptions* of smaller hyperedges by larger facets. Figures 3c,d illustrate the difference between hyperedge and facet statistics for another co-authorship data set (Physical Review D journal). Like in Fig. 3a, there is significant difference at the head of the size distributions, implying a significant frequency of subsumptions. Also, in both Fig. 3b and d, the tails of the facet degree distribution are shorter than those of the respective hyper-degree distributions. This can be attributed to the subsumption of many small hyperedges at the high-degree nodes, thus reducing the facet degree compared to their hyperedge degrees.

**Figure 4:**
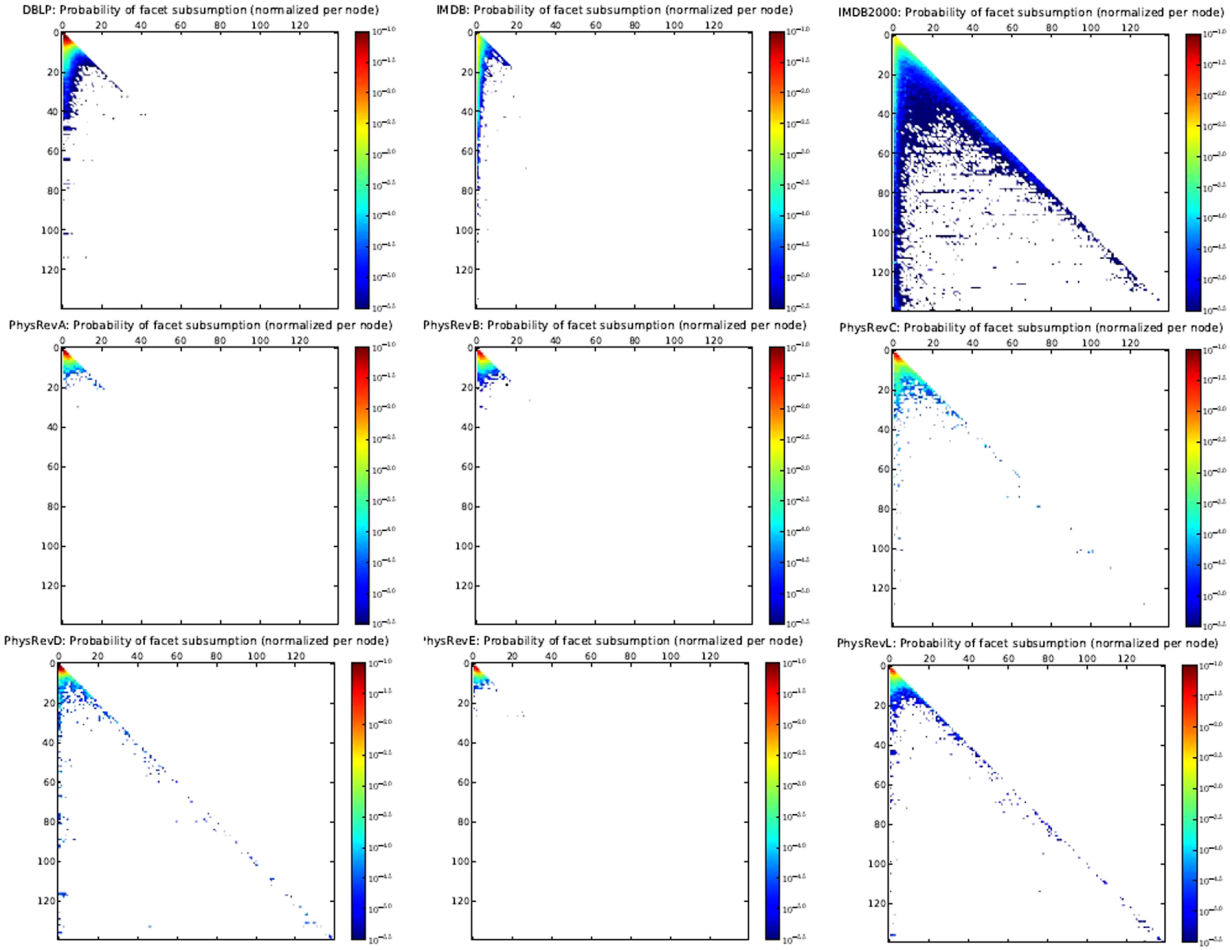
Subsumptions in collaboration network data (in each plot, rows and columns correspond to sizes of *subsuming* and *subsumed* facets, respectively): (**a**) DBLP, (**b**) IMDB (only regular movies and cast), (**c**) IMDB 2000 (all movies, cast, and crew year 2000 onwards), (**d**) Phys Rev A, (**e**) Phys Rev B, (**f**) Phys Rev C, (**g**) Phys Rev D, (**h**) Phys Rev E, (**i**) Phys Rev Letters.

We now examine the nature of subsumptions in real collaboration datasets more closely. In Fig. 4, we plot nine real collaboration data sets, the number/percentage of subsumptions of facets of size *i* by facets of size *j* (where *j* ≥ *i*). Obviously, this is a lower triangular matrix where the rows indicate sizes of *subsuming* facets and columns indicate sizes of *subsumed* facets.

We observe that the nature of subsumptions varies across the nine data sets. In DBLP, IMDB (with regular movies and cast only), Phys Rev A, Phys Rev B, and Phys Rev E, small facets are subsumed (first few columns). This is intuitively expected, since it is likely that smaller subsets of a collaboration are also valid collaborations.

In Phys Rev D, Phys Rev L and Phys Rev C, there is a strong subsumption presence both on and off the diagonals. These subsumption events model scenarios where a significant number of individuals are collaborating on a task and the exact set of individuals collaborate again, with perhaps a few additional collaborators such as new graduate students joining a lab. These situations likely arise from the large endeavors typical of experimental physics where very large collaborations of laboratories result in a paper, as evidenced by Phys Rev D and L. In fact, for Phys Rev D, the diagonal has non-trivial mass even for collaboration sizes of 500, which have not been shown here.

Finally, the IMDB data set that includes both cast and crew of movies (c) is a class apart in the above trend taken to a much larger magnitude. This is because a core crew tends to get utilized by a director in multiple movies. As seen from these figures, such events occur often in reality, hence subsumption is an important issue to address when modeling the structure of the underlying global collaboration network.

## Generative Growth Models for Networks Induced by Global Collaboration Relationships

Generative network growth models have received great interest over the past 15 years for classical (binary) graphs. The most prominent growth model has been *preferential attachment*, which has been demonstrated to result in graphs with node degrees following power law distributions^14^, i.e. *k* ^−*γ*^, where $\gamma \in \mathrm{[2,}\,\mathrm{3]}$. Examples of other network growth models that have received attention are small-world models^15^, densification models^16^, and duplication models^17^, to name some.

In contrast, there has been a limited amount of work on generative models for group collaboration structures. In addition to the *node degree* distribution, which has been a focal metric for classical network growth models, for collaboration structures more complex than graphs, the hyperedge size distribution is key. Hebert-Dufresne *et al*.^6^ proposed a generative algorithm called Structural Preferential Attachment (SPA) by progressively growing a hypergraph based on parameters that depend on the power law exponents of hyperedge size and degree distributions (their assumption was that both obey power law distribution). In SPA, *two* free probability parameters are simultaneously controlled to generate new structures and attachment points in the current network in order to simultaneously match the tails of both the hyperedge degree and size distributions. However, as Fig. 3 suggests, this is not accurate for many collaboration *relation* structures, particularly for the size distributions. More importantly, *structure-based* growth models^2, 6^ do not focus on modeling the structure of the underlying global collaboration network–instead they model the “artifacts” of collaboration, i.e., hyperedges.

To generate real world collaboration relation structures, we propose a generative facet-by-facet growth model based on preferential attachment, not based on an individual *X*’s *degree*, that is, how many people has *X* collaborated with over a period of time, but on *X*’s *facet degree* which measures how many maximal collaborations or *facets* has *X* been involved in. The basic intuition behind this is the following: individuals who are comfortable being part of several distinct collaboration endeavors are likely to attract more collaborators than the individuals who are happy participating in a fewer number of collaboration endeavors (albeit with several collaborators).

We assume that the facet size distribution is given to us and our primary aim is to generate a random simplicial complex that closely matches the ground truth distribution for facet degrees. We do this because the facet size distributions are often *non-power law* and even *non-monotonic*, particularly near the head of the distribution as in Fig. 3a and c, thus not obeying trends of typical distributions generated by random growth models. On the other hand, facet degree distribution in Fig. 3b has monotonic behavior and is power law with exponential cutoff, implying that it might be possible to obtain it more accurately via random growth models. Accordingly, our facet-based generative model takes as input the facet size distribution and grows the *collaboration simplicial complex* one (transient) facet at a time. During this process, a large facet may *subsume* a smaller (transient) existing facet since we are interested in capturing the fundamental structure of collaboration.

In related work, we first acknowledge the early work^1^ which empirically points out the basic properties of scientific collaboration network structures. Over the past decade, a significant number of researchers have studied structures representing collaboration artifacts. Liu *et al*.^8^ have proposed a preferential-attachment based growth model for “affiliation networks”. However, this is only a qualitative study without any theoretical analysis. Some authors have considered the artifacts of collaboration^3^, and have provided analytic results for only very special cases where the collaborative outputs are fixed size. This was also the assumption elsewhere^18^ with the authors studying the evolution processes for the network of scientific collaboration artifacts. On the other hand, an empirical study focused on a very specific scientific collaboration network has been provided^5^; and a modified preferential attachment algorithm has been considered^4^, again for collaboration artifacts along with numerical results. Our work goes beyond these models to provide both a theoretically sound treatment, and accounts for the key phenomenon of subsumption that occurs in various collaborative endeavors. In other work focused on going beyond pairwise relationships, properties of random tripartite hypergraphs have been studied^19^. Also, Wu *et al*.^20^ have recently proposed a simplicial complex generation model but with a significantly different goal of characterizing the growing geometry of networks. Similarly, Bianconi *et al*.^21^ consider the Network Geometry with Flavor (NGF) and provide a quantum mechanical description. Courtney *et al*.^22^ consider a configuration model treatment for simplicial complexes of a given dimension. On the other hand, Lambiotte *et al*.^23^ propose a growth model based on the copying mechanism and study the resulting phase transition behavior. Very recently, growth models for collaboration artifacts have been considered^9^, but they address neither the success of matching the size distribution nor the subsumption phenomena, which is a focal point of this paper.

## Results

GENESCs has several distinctions from other works that have proposed PA based generative growth models for hypergraphs^6, 8^. Since we are interested in modeling the global collaboration relation and not its artifacts, in our model the *hyperedges* are subsumed to yield *facets*, thus preserving the core structure underlying the participation of various individuals in a collaboration. We do not assume that the size of such collaborative structures is power law distributed. In fact, this is not the case for several collaboration networks, especially near the head, i.e., small collaborations. Accordingly, GENESCs takes *as input* the distribution of facet sizes and average facet density (i.e., average number of collaborations per node) to generate a collaboration relation using a variant of PA.

The dynamics of GENESCs is distinct from classical PA in two ways. First, the structural unit of growth in each time step in our setting is a (transient) *facet* which could contribute one or more nodes to the simplicial complex. In contrast, in classic PA, one node is added in each time step. Secondly, in classical PA new nodes are always added to the network, whereas in our case, some nodes in the newly generated facet may be merged with existing nodes; moreover, new facets may *subsume* older facets or *be subsumed* by older facets. This is consistent with how large collaboration networks grow–new endeavors consist of both existing individuals and new individuals.

It is well known that preferential-attachment (PA) based network growth methods result in power law node degree distributions for classical graphs^14^. Other more general variants of preferential attachment have been analyzed extensively as well^24^. We show below that the growth model behind GENESCs results in SCs with power law distributed facet degree. We also compute the power law exponent as a function of input parameters such as average facet size and average facet density, and show that it matches real world collaboration network data sets well.

**Theorem 0**.**1** (**Facet degree properties**) *Let the current number of nodes and facets in the simplicial complex* (*SC*) *be denoted by n and f*, respectively. Also let *k*_*i*_ denote the facet degree of node *i*, and facet sizes are given by ${s}_{j},j\in \mathrm{[1},f]$. If the average facet density (the ratio of the number of facets to the number of nodes) is *c* and average facet size is *s*, GENESCs generates a random simplicial complex whose facet degree distribution is power law with exponent $\alpha \mathrm{=2}+\frac{1}{c\,s-1}$.

**Proof:** In Supplemental Information Section 2.                          □

Note that the denominator of the exponent *cs* − 1 is strictly positive for simplicial complexes of interest. This is because $cs=\frac{sf}{n}=\frac{{\sum }_{j=1}^{f}{s}_{j}}{n}=\frac{{\sum }_{i=1}^{n}{k}_{i}}{n}$. Since ∀*i* : *k*_*i*_ ≥ 1, for any non-degenerate SC that is not a disjoint union of full dimensional simplexes, ${\sum }_{i\mathrm{=1}}^{n}{k}_{i} > n$, and therefore *cs* − 1 > 0.

The quantity *cs* can be interpreted as the average number of *collaborations* of a node.

### Performance evaluation of GENESCs

We measured the quality of the distribution generated by GENESCs using the Kolmogorov-Smirnov distance *D*_*KS*_ and the Total Variation distance *D*_*TV*_ between real data distributions *p*(⋅) and the generated distributions *q*(⋅) for both facet sizes and facet degrees: ${D}_{KS}(p||q)={{\rm{\sup }}}_{x}|p(x)-q(x)|$, and ${D}_{TV}(p||q)=\frac{1}{2}{\sum }_{x}|p(x)-q(x)|.$

Note that the facet sizes in GENESCs are generated using the facet size distribution of real collaboration data sets, where the effect of subsumptions has already been incorporated in the first place. We observed that GENESCs yields *D*_*KS*_ ≈ 0 and *D*_*TV*_ ≈ 0 for facet sizes, confirming the fact that subsumption events are rare if one is drawing random collaboration structures from the *facet* size distribution instead of the *hyperedge* size distribution. Analytic arguments for this phenomenon are presented in Supplemental Information Section 3.

**Figure 5:**
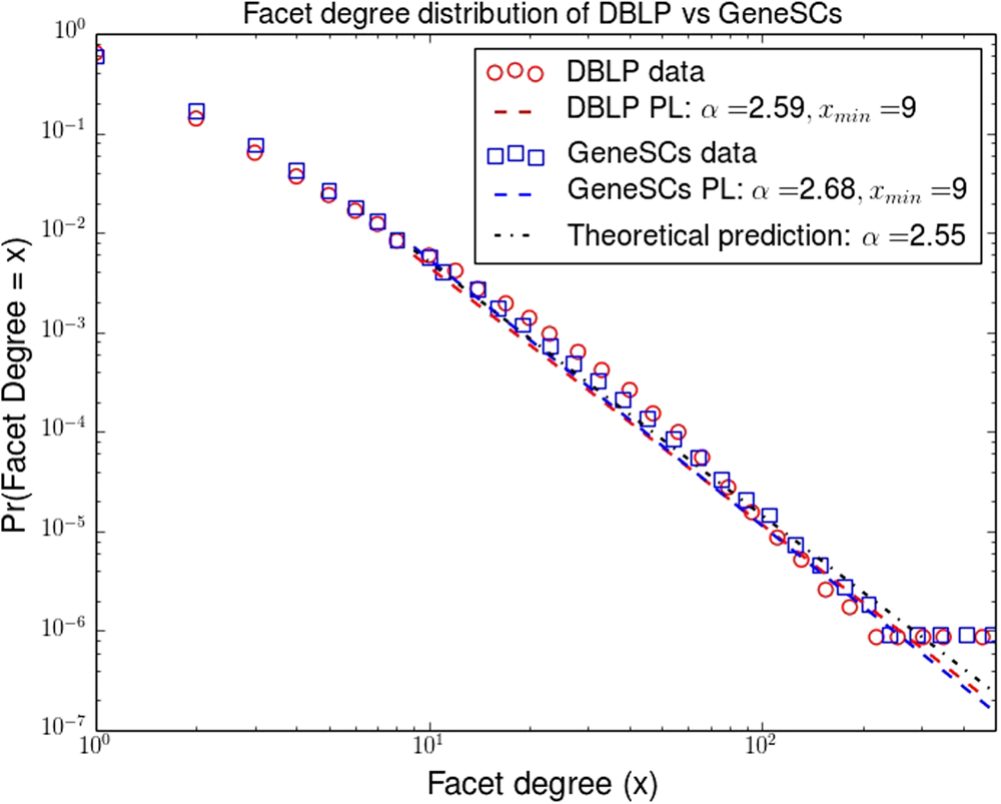
Performance of GENESCs vs. real DBLP collaboration data. Since longitudinal DBLP data from 1936 to 2011 was available, we used GENESCs parameters *c* = 0.2447, *β* = 1.0858 which accurately fit the growth of the simplicial complex during that period of time (see Methods section). We find that *D*_*KS*_ = 0.023, *D*_*TV*_ = 0.054. Tail behaviors of various power law curves were superimposed on logarithmic binned data. Since our theoretical calculations consider a simplified growth model with a single parameter, the final facet density *c* and *β* = 1, we used *c* = 0.82 (ratio of final number of facets, *f* = 900 *k* to final number of nodes, *n* = 1.1 *M*) and average facet size *s* = 3.44 to compute the analytical prediction of the power law exponent of GENESCs, *α* ≈ 2.55. The noisy tails are due to a small number of samples, which is typical of logarithmic binning.

Figure 5 illustrates the performance comparison of GENESCs with respect to real DBLP publication data and the theoretical prediction of Theorem 0.1 as far as the facet degree distribution is concerned. It can be observed that GENESCs matches well the characteristics of both the real facet degree distribution and the theoretical prediction.

**Figure 6:**
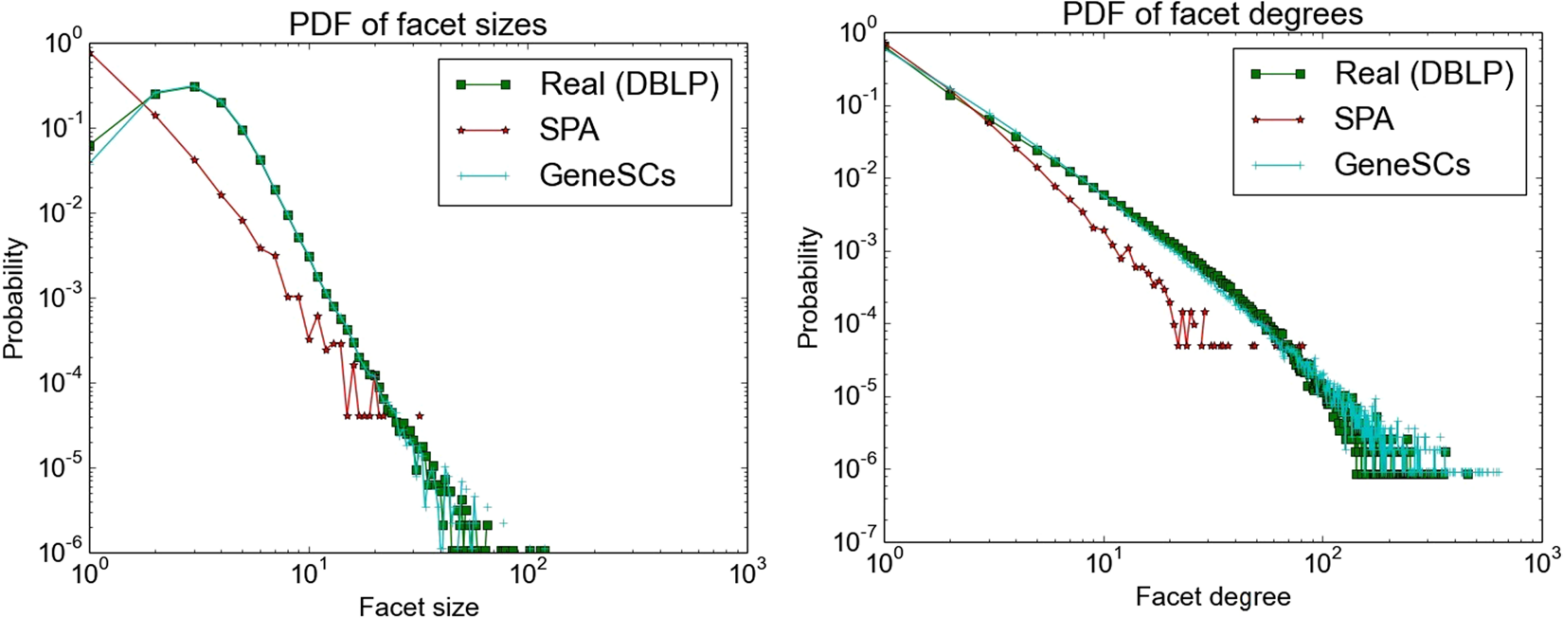
Comparison with SPA algorithm for DBLP, (**a**) Facet sizes: *D*_*KS*_ = 0.012, *D*_*TV*_ = 0.0246 for GENESCs, *D*_*KS*_ = 0.36, *D*_*TV*_ = 0.72 for SPA. (**b**) Facet degrees: *D*_*KS*_ = 0.023, *D*_*TV*_ = 0.054 for GENESCs, *D*_*KS*_ = 0.036, *D*_*TV*_ = 0.094 for SPA.

Figure 6 compares the relative performance of the Structured Preferential Attachment (SPA) algorithm^6, 7^ with GENESCs. When applying SPA, first the parameters that best fit DBLP’s hyperedge degree and size distributions are obtained and used for hyperedge generation. Then, we have inflicted subsumptions on that data and plotted it. It can be observed from Fig. 6(a) that SPA, which is designed to generate pure power law distribution for both hyperedge sizes and hyper-degrees, is unable to match the real facet size distribution of the DBLP data set (*D*_*TV*_ = 0.72). It is also unable to closely match the facet degree distribution of DBLP (*D*_*TV*_ = 0.094), especially near the heavy tail. Moreover, it has a markedly different slope. In contrast, since GENESCs samples the facet size distribution, it is able to yield a close match to (obviously) that distribution (*D*_*TV*_ = 0.023) (the minor difference is due to the occurrence of some subsumptions at low sizes), and the facet degree distribution (*D*_*TV*_ = 0.054).

**Figure 7:**
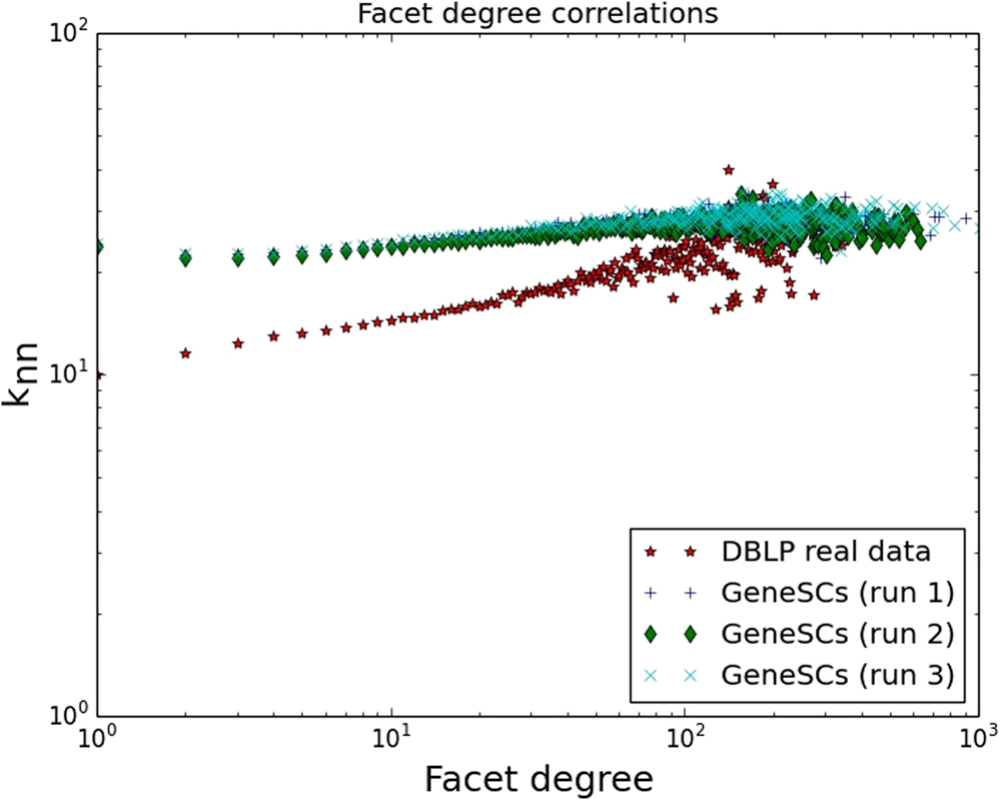
Facet degree correlations for DBLP dataset.

While we have primarily focused thus far on demonstrating the success in matching facet degree and facet size distributions, here we also provide more advanced network metrics that confirm the success of GENESCs. First among these is *neighborhood facet degree correlation*. We adapt the node degree correlation metric *k*_*nn*_, defined in^25^ to simplicial complexes as ${k}_{nn}(k)={\sum }_{k^{\prime} }k^{\prime} P(k^{\prime} |k)$, where *P*(*k* ^′^|*k*) is the probability that a node with facet degree *k* will have a neighbor (in the simplicial complex) with facet degree *k*′. As demonstrated in Fig. 7 for the DBLP dataset, we find that both the dataset and the random simplicial complexes produced by GENESCs have extremely good match at the tail, and both real and generated networks are *assortative*, i.e., high degree nodes tend to be surrounded by high degree nodes as is typically observed in scientific collaboration networks^26^. This can be concluded from the fact that the *k*_*nn*_(*k*) function has a positive slope with increasing facet degree *k*.

**Figure 8:**
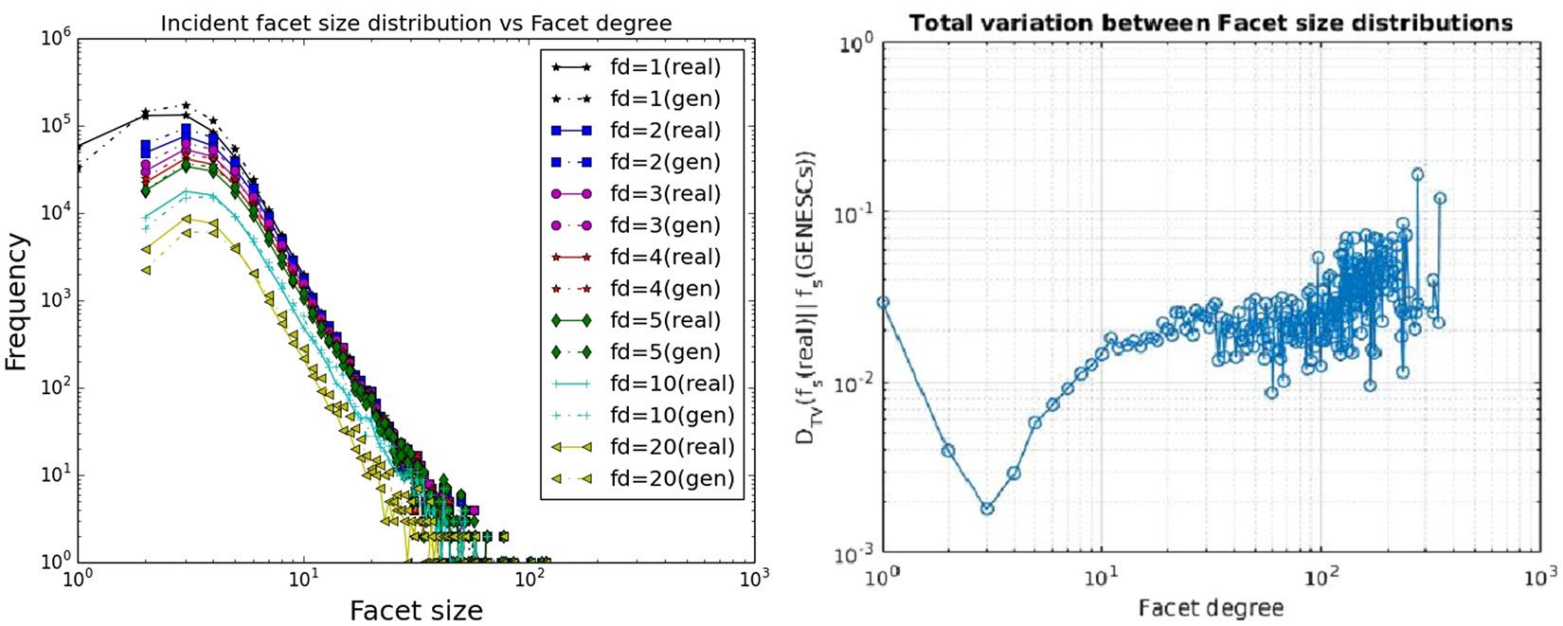
Incident facet sizes on various facet degrees: (**a**) Distributions of sizes of facets incident on nodes of different facet degrees (**b**) Total variation distances of facet size distributions conditioned on facet degree.

In addition, Fig. 8 demonstrates that facet size distributions conditioned on a fixed facet degree resulting from GENESCs closely follow the real dataset. The figure also points out that a facet size of one can only occur for facet degree one in a simplicial complex–for an isolated facet (which corresponds to a single node).

## Discussion

In this paper, we proposed GENESCs, a generative model for collaboration structures modeled as simplicial complexes (SC). SCs are different from graphs since they have two different dimensions for growth (facet size and facet degree), whereas graphs only have one (node degree), since all edges have identical sizes. SCs are also different from hypergraphs, which do not have the subsumption property. While hypergraphs are good for modeling artifacts of collaborations, e.g., papers and movies, SCs are more appropriate for succinctly modeling the inherent structure of the collaboration relationship, i.e., who all have collaborated with each other. This distinction is key because subsumptions are very common in real-world collaboration networks.

The facet size distribution constrains the facet degree distribution to an extent. Leveraging this observation, GENESCs takes facet size distributions of real world collaboration networks as input and efficiently generates random SCs with facet degree distributions matching those corresponding to the real data. Our theoretical analysis is shown to accurately predict the statistical properties of SCs generated by GENESCs in its pure form. For collaborations that have characteristics such as very heavy tails of facet sizes or non-power law popularity distributions of participants as collaborators, appropriate modifications to the preferential attachment step of GENESCs (namely, clamping and uniform-hybridization) yields good intuitively sound results.

## Methods

### GENESCs: A Facet-based Preferential Attachment Model

GENESCs takes as input the facet size distribution *z*(*s*) of a real data set (where *s* is the facet size with 1 ≤ *s *≤ |*V*|) and generates a random simplicial complex (*V*, *F*) modeling that data set with facet degree distribution *p*_*k*_ (where *k* denotes facet degree).

It has been observed from recent studies on simplicial complex models of collaboration networks such as DBLP and IMDB^13^ that in a simplicial complex *S* = (*V*, *F*), there exists a relationship between the number of facets |*F*| and number of nodes |*V*|:|*F*| = *c*|*V*|^*β*^. Parameters *c*, *β* can be estimated if data about the longitudinal evolution of the collaboration network is available. We exploit this relationship to steer GENESCs to generate simplicial complexes that match real world datasets. If longitudinal data is unavailable, we assume that *β* = 1; then *c* is the *facet density*.

The algorithm (shown in pseudo-code form in Supplemental Information Section 4) grows the simplicial complex *S* by adding one randomly generated (transient) facet at a time, decides points of attachment in *S*, and checks for subsumption events. Note that before the addition of a (transient) facet indexed by integer *f* and denoted by *F*_*f*_, the state of the simplicial complex is denoted by *S*(*f*) = (*V*(*f*), *F*(*f*)).

GENESCs is computationally efficient. We store the Simplicial Complex in a sparse matrix with |*F*(*f*)| rows and the number of columns is a maximum facet size encountered so far. The biggest computational bottleneck is the PA step in line 11. To speed up the computation, we use the following identity relating the sum of facet degrees to the sum of facet sizes, are easy to update after every step.

1 $$\sum _{u\in V(f)}{f}_{d}(u)=\sum _{f\in F(f)}|f|$$

Here, *f*_*d*_(*u*) is the facet degree of node *u* and |*f*| is the size (number of nodes) in facet *f*. Another source of speedup is computing subsumptions by solving a problem of matching two small substrings corresponding to facets (small relative to |*V*|) as shown in Supplemental Information Section 4.

### Variants of GENESCs: Smoothed Preferential Attachment

While GENESCs performs notably well for DBLP using pure preferential attachment (PA) on facet degrees, it is unable to generate the facet degree distributions for collaboration networks such as Physical Review D (PRD) and IMDB. Hence, we propose two variants of PA to address this drawback.

#### Clamped Preferential Attachment

While PA is likely to be a basic force behind collaboration network formation, some domains leverage other peculiar behaviors while forming large collaborative structures. For example, the circumstances and motives behind the formation of “organic” academic collaborations are typically different from those driving the formation of collaborations in entertainment or artistic fields (e.g. movies). Even in academia, the way research is conducted highly depends on the particular sub-field, as theoretic and experimental communities have different processes of forming groups and performing collaborative research. Hence, it is not surprising that GENESCs is not a one-size-fits-all solution. For instance, the maximum facet degree of the generated SC tends to exceed the maximum facet degree of the real dataset. Moreover, the facet degree distributions of the generated and real SC have an unacceptably high total-variation distance, *D*_*TV*_ = 0.2.

Reflecting on this behavior, we posit the following hypothesis for real networks: individuals typically do not categorize the popularity of other individuals precisely by the number of connections; rather, they might make better assessment regarding people with *similar* popularity, i.e., they have a coarser perception, particularly for very popular individuals. This is indeed an issue with collaborations in experimental physics, which tend to publish in Physical Review D. There are several papers reporting results from large experiments with very large sets of co-authors (See Fig. 3). In such networks, a coarse grained view of popularity is likely to better explain network growth.

To model this hypothesis, we modify GENESCs in the PA step (particularly defined on line 11 of Algorithm GENESCs). More precisely, rather than using the exact facet degree, we *clamp* the facet degrees of each node in order to smooth the perception of popularity of the very high degree nodes. In the most basic form, one can achieve this as follows: ${f}_{cld}(u)=\,{\rm{\min }}({f}_{d}(u),{F}_{d}^{clamp}),$ where *f*_*d*_(*u*) is the actual facet degree of node *u*, ${F}_{d}^{clamp}$ is the maximum clamp value and *f*_*cld*_(*u*) is the clamped facet degree which is input to the PA step of GENESCs.

While this method classifies many very popular individuals as *popular*, it is likely to be limited, since too much granularity is lost. Consequently, we actually use a softer mapping (unlike the step function mentioned earlier) as:

2 $${f}_{cld}(u)=\{\begin{array}{cc}{f}_{d}(u), & \,if\,{f}_{d}(u) < \alpha \,{f}_{d}^{max}\\ \sqrt{\alpha \,{f}_{d}(u)\,{f}_{d}^{max}}, & \,if\,{f}_{d}(u)\ge \alpha \,{f}_{d}^{max}\end{array}$$

where *α* is a design parameter, and ${f}_{d}^{max}$ is the maximum of actual facet degrees at the current step, i.e., ${f}_{d}^{max}={max}_{u}\,{f}_{d}(u)$.

**Figure 9:**
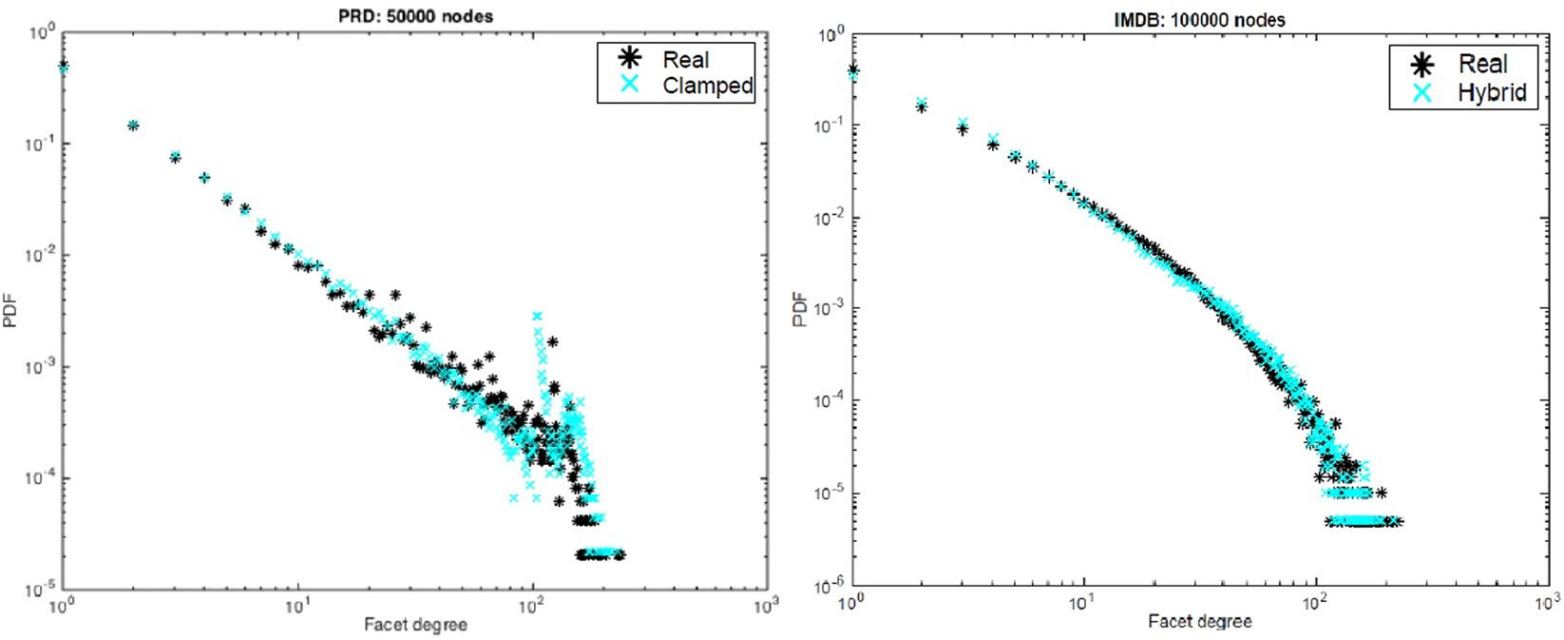
Variants of GENESCs: (**a**) Clamped PA: Phys Rev D data (*α* = 0.25): *D*_*TV*_ = 0.035 (**b**) Hybrid PA and uniform attachment: IMDB data (*T* = 12, *α* = 0.25):*D*_*TV*_ = 0.04.

Effectively, this assignment *still* differentiates among the *very popular* nodes, but smooths their relative popularity values considerably. After this mapping is performed, GENESCs operates using these alternative facet degrees. For the sake of completeness, line 11 of Algorithm GENESCs is replaced by the following modified-PA rule: $p(u)=\frac{{f}_{cld}(u)}{\sum _{u\in V}{f}_{cld}(u)},$ with *f*_*cld*_(*u*) obtained through (2). Here we note that while modifying standard PA has been considered in^3^, which propose a nonlinear preferential attachment by replacing node degree *f*_*d*_(*u*) by *f*_*d*_(*u*)^*β*^ for each node in the connection process with some given *β*, our clamped PA defined by the mapping in (2) is significantly different and also depends on many distinct parameters as *α* and ${f}_{d}^{max}$. Figure 9a shows that the clamped PA can yield a close match to the facet degree distribution in the PRD data set, including the spikes at the tail.

#### Hybrid Uniform and Preferential Attachment

Another behavior that we intuitively expect in building collaboration structures is that it is rarely the case that every member of a large collaboration is well-known. In other words, typically a limited number of collaborators are popular and the remaining ones are not really well-known or popular. For instance, consider a movie cast. It is often the case that only a small subset (e.g. 10–15) of the whole movie cast constitutes well-known actors, which have more screen time and central roles (and hence are immediately identifiable by an average movie goer), while the rest are mostly figures with minor side roles.

Based on the above intuition, particularly for IMDB style collaborations, we propose the following variant to GENESCs: At line 8 of the algorithm, out of *mergev* connections to the existing simplicial complex, only the first *T* (or *min*(*mergev*,*T*)) nodes to merge are determined by applying PA on the facet degrees. The remaining *mergev* − *T* nodes (assuming *mergev* > *T*) to be merged are selected *uniformly* at random, regardless of their facet degrees. We observe that this *preferential-uniform hybrid* GENESCs along with clamped facet degrees for the PA sub-routine provides a much better match to the distributions of actual IMDB dataset, thus verifying our qualitative intuitions. This can be observed in Fig. 9b.

While the idea of combining preferential attachment and uniform attachment has been considered before^27^, it has been done so only for graphs. Moreover, the latter approach *probabilistically mixes* the two methods for every connection, in contrast to our threshold-based approach for generating SCs.

## Electronic supplementary material

Supplementary Information
