# Supplementary material for: Generative Models for Global Collaboration Relationships
Source: Sci Rep. 2017 Sep 11;7:11160. doi: 10.1038/s41598-017-10951-5 (PMC5593992; doi:10.1038/s41598-017-10951-5)
Supplement: Supplementary file 1 — Supplementary Information [file 41598_2017_10951_MOESM1_ESM.pdf]

Supplementary Information for Manuscript "Generative Models for Global Collaboration Relationships"

Authors: Ertugrul Necdet Ciftcioglu, Ram Ramanathan, Prithwish Basu

1 Simplicial Complexes Illustration

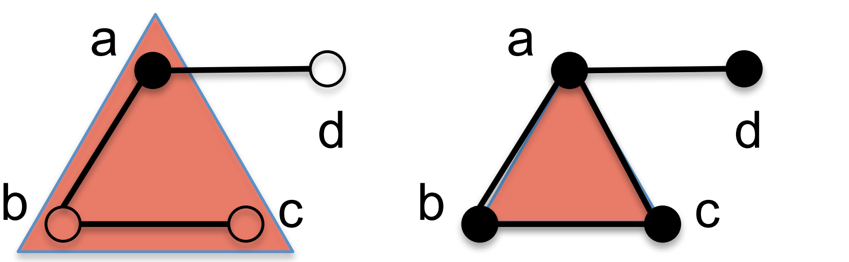

Figure 1. Hypergraph vs. Simplicial Complex: In the hypergraph, a dark node denotes an author who has written least one paper as a sole author. In a simplicial complex, all nodes are dark since being dark just means than the corresponding author has written at least one paper, with zero or more collaborators (0-simplex). In this example, each 0-simplex belongs to a 1-simplex (edge) and in case of a, b, and c, also a 2-simplex.

2 Proof of Theorem 0.1

We use mean field arguments in this proof. Let the current number of nodes and facets in the simplicial complex (SC) be denoted by n and f, respectively. Since c is the facet density, we have $c = \frac{f}{n}$, at least when the SC has grown large in size. At the current time step, a new facet arrives into SC and the facet count becomes $f+1$. Simultaneously, the node count is expected to increase to $\frac{f+1}{c} = n + \frac{1}{c}$ (Note that for the purpose of clarity, throughout the analysis we ignore the effects of rounding to integer values for some of the variables.)

Let $p_k(f)$ be the fraction of nodes in SC with facet degree (fdegree) k when there are f facets in the SC. If node i has fdegree k_i, then the PA step in GeneSCs will merge a node from the newly arriving facet into node i with probability $p_i = \frac{k_i+a}{\sum_{i=1}^{n}(k_i+a)} = \frac{k_i+a}{\sum_{i=1}^{n} k_i + an}$, where a is the initial attractiveness parameter[1]. It can be observed that if facet sizes are given by $s_j, j \in [1, f]$, we have $\sum_{i=1}^{n} k_i = \sum_{j=1}^{f} s_j = fs$. Therefore, $p_i = \frac{k_i+a}{fs+an} = \frac{k_i+a}{cns+an} = \frac{k_i+a}{(cs+a)n}$.

When the $(f+1)$-th facet of average size s is added to SC, on average the number of new nodes that are added to SC are $\frac{f+1}{c} - \frac{f}{c} = \frac{1}{c}$. Therefore, GeneSCs attempts to merge $s - \frac{1}{c}$ nodes (on average) in the new facet with old nodes in SC by performing PA independently for each such node.

Just like in the regular PA for graphs, the probability of more than one node getting merged with a single old node in SC goes is vanishingly small as $n, f \to \infty$, hence we assume that each of the $s - \frac{1}{c}$ nodes in the new facet get merged to distinct nodes in SC with high probability (Please see Lemma 3.1). Since there are $np_k(f)$ nodes in the SC with fdegree k, the expected number of new collaborations (this new facet) picked up by all nodes of fdegree k in SC as a result

of the addition of the new facet is given by $np_k(f)\frac{k+a}{(cs+a)n}(s-\frac{1}{c}) = p_k(f)\frac{k+a}{cs+a}(s-\frac{1}{c})$. Thus, the expected number of nodes in SC whose fdegree becomes $k+1$ as a result of the facet arrival is given by $p_k(f)\frac{k+a}{cs+a}(s-\frac{1}{c})$.

We observe that for each node with fdegree $k-1$ that gets merged with the new facet, the number of collaborations increases by one, thus increasing their fdegree to k. Applying the above reasoning, the expected number of such collaborations is thus $p_{k-1}(f)\frac{k-1+a}{cs+a}(s-\frac{1}{c})$. Also, the expected number of nodes with fdegree k after the addition of the new facet is $(n+\frac{1}{c})p_k(f+1) = \frac{f+1}{c}p_k(f+1)$, since there are $n+\frac{1}{c}$ nodes in the SC at this stage.

It can be shown that the facet count increases by one at least for large SCs, since under GENESCS the probability of a newly arriving facet subsuming an existing facet or getting subsumed becomes vanishingly small as $n, f \to \infty$. See Lemmas 3.2, 3.3, 3.4, 3.5, 3.6, and Remark 3.1 in Appendix 3 for details. The fact that the amount of subsumption resulting from GENESCS is small is desirable since we utilize the facet size distribution $f(s)$ as an input parameter, and all the hyperedge to facet subsumption is already captured in $f(s)$. Therefore, one can set up the "master equation" that results from the conservation of collaboration counts after the addition of the $(f+1)$-th facet into SC:

$$(n+\frac{1}{c})p_k(f+1) = np_k(f) + \frac{k-1+a}{cs+a}(s-\frac{1}{c})p_{k-1}(f) - \frac{k+a}{cs+a}(s-\frac{1}{c})\,p_k(f) \tag{1}$$

Substituting $n=\frac{f}{c}$ into Eq. (1), we get the master equation as a function of f alone.

$$(f+1)p_k(f+1) = fp_k(f) + \frac{(k-1+a)(cs-1)}{cs+a}p_{k-1}(f) - \frac{(k+a)(cs-1)}{cs+a}\,p_k(f) \tag{2}$$

Note that for $k=1$ (the lowest fdegree in SC), Equation (2) is not accurate since there is no dependence on $p_{k-1}(f)$. Instead, the new facet has a contribution of $\frac{1}{c}$ new nodes of fdegree 1, on average. We reflect this in the following equation for $k=1$:

$$(f+1)p_1(f+1) = fp_1(f) + 1 - \frac{(1+a)(cs-1)}{cs+a}\,p_1(f) \tag{3}$$

Assuming that $p_k(f)$ converges to p_k when $f\to\infty$ for all k, we rewrite Equation (3) and substitute $g=cs$ to get:

$$p_1 = 1 - \frac{(1+a)(g-1)}{g+a}\,p_1 \Rightarrow p_1 = \frac{g+a}{g(1+a)+g-1}$$

Performing similar transformations to Equation (2), we get

$$p_k = \frac{k-1+a}{k+\frac{g(1+a)}{g-1}}\,p_{k-1} = \frac{(k-1+a)\cdots(1+a)}{(k+\frac{g(1+a)}{g-1})\cdots(2+\frac{g(1+a)}{g-1})}\cdot\frac{g+a}{g-1+g(1+a)}$$

Using the basic recurrence for Gamma functions $\Gamma(x+1) = x\Gamma(x)$, we have the identity $\frac{\Gamma(x+n)}{\Gamma(x)} = (x+n-1)(x+n-2)\cdots x$. Using this identity, we have:

$$\begin{aligned} p_k &= \frac{\Gamma(k+a)\,\Gamma(2+\frac{g(1+a)}{g-1})}{\Gamma(1+a)\,\Gamma(k+1+\frac{g(1+a)}{g-1})}\cdot\frac{g+a}{1+\frac{g(1+a)}{g-1}} = \frac{\Gamma(k+a)\,\Gamma(1+\frac{g+a}{g-1})}{\Gamma(k+1+\frac{g(1+a)}{g-1})}\cdot\frac{\Gamma(2+\frac{g(1+a)}{g-1})}{\Gamma(1+a)\,\Gamma(1+\frac{g+a}{g-1})}\times\frac{g+a}{g-1+g(1+a)} \\ &= \frac{B(k+a,1+\frac{g+a}{g-1})}{B(1+a,1+\frac{g+a}{g-1})}\cdot\frac{g+a}{g-1+g(1+a)}, \end{aligned} \tag{4}$$

where $B(x,y) = \frac{\Gamma(x)\Gamma(y)}{\Gamma(x+y)}$ is the Beta function.

For large x, $B(x,y)$ exhibits power law behavior; specifically, $B(x,y)\approx x^{-y}\Gamma(y)$. In Equation (4), the only term that is dependent on k is $B(k+a,1+\frac{g+a}{g-1})$. Applying the aforementioned power law approximation for large k, we get:

$$p_k \sim (k+a)^{-(1+\frac{g+a}{g-1})} \sim k^{-(1+\frac{g+a}{g-1})} = k^{-(2+\frac{1+a}{g-1})} = k^{-(2+\frac{1+a}{cs-1})}$$

Therefore, the facet degree of a large SC generated by GENESCS is power law distributed with exponent $2+\frac{1+a}{cs-1}$. Since we set the attractiveness parameter $a=0$ in the default mode of GENESCS, the result follows.

3 Characterizing the Probability of Facet Subsumption

In this appendix, we consider various situations in which a newcomer facet subsumes one or more facets in the existing SC when following the rules of GENESCS. In the following lemmas, we show that the probabilities of such subsumptions become vanishingly small as the SC grows in size. Similar reasoning can be applied for the reverse case, where a newcomer facet is subsumed by an existing facet in the SC.

Lemma 3.1 *The probability of more than one node getting merged with a single existing node in the SC generated thus far becomes vanishingly small as $n, f \to \infty$, where n and f denote the number of nodes and facets in the SC, respectively.*

Proof: Assume that at the current facet-addition step f of GENESCS (at this step, SC is supposed to have f facets), the newly generated facet of (average) size s is to be merged with the existing SC at $m \approx s - \frac{1}{c}$ nodes. Let Z_k be a random variable denoting the number of merges of an existing node (say, X) of facet degree k with one or more distinct nodes $Y_1, Y_2, \ldots$ of the incoming facet. For large enough f, the probability that Y_i gets merged into X is $\frac{k}{fs}$ following the Preferential Attachment rule (X has facet degree k and there are fs nodes in the current SC). Assuming m independent Bernoulli trials for merges (this is reasonable in the large network limit as in [1]), the probability of merging l times with X can be expressed as a Binomial random variable. Accordingly, the aggregate probability of having l merges is $\binom{m}{l}(\frac{k}{fs})^l(1-\frac{k}{fs})^{m-l}$.

$$P(Z_k > 1) = \sum_{l=2}^{m} \binom{m}{l} \left(\frac{k}{fs}\right)^l \left(1 - \frac{k}{fs}\right)^{m-l} \tag{5}$$

$$\begin{aligned} P(Z_k = 2) &= \frac{m(m-1)}{2} \left(\frac{k}{fs}\right)^2 \left(1 - \frac{k}{fs}\right)^{m-2} && (6) \\ &< \frac{s(s-1)}{2} \left(\frac{k}{fs}\right)^2 \left(1 - \frac{k}{fs}\right)^{m-2} && (7) \\ &\leq \frac{k^2}{2f^2} = O\left(\frac{k^2}{f^2}\right), && (8) \end{aligned}$$

where in (7) we have used the well-known Bernoulli inequality $(1-x)^a \leq 1 - xa$ for $x < 1$, since $\frac{k}{fs} \to 0$ as $f \to \infty$. It can be readily shown that $P(Z_k = l) = O(\frac{k^l}{f^l})$, hence the probability of multiple edges merging to a given node with degree k is $O(\frac{k^2}{f^2})$. □

We propose the *supernode method* to establish upper bounds on the subsumption probability. In order to investigate whether a given facet is subsumed by the new-coming facet at step f, we form a *virtual supernode* which models the nodes of the facet jointly as shown in Figures 2-3. More specifically, the facet degree of the supernode is assigned to be the sum of the facet degrees of the individual nodes.

Lemma 3.2 *For any scenario where the facet dimension is bounded, the probability of subsumption is $O(\frac{1}{f^2})$, and thus negligible in large SCs.*

Proof: Consider the probability of a facet being subsumed. For instance, when the facet under consideration is an edge (i, j), this means two distinct nodes s and t of the m nodes of the newcomer facet F_f to be merged into the existing network are specifically merged to i and j. This can happen as ($s \to i$ and $t \to j$) or ($s \to j$ and $t \to i$).

Now define the supernode K comprised of edge (i, j). Let us consider the probability that K is selected for merging to more than one node of F_f (Fig. 4). To analyze this, K can be treated as an ordinary node with degree $d_i + d_j$. From Lemma 3.1, with preferential attachment, the probability of any node v with degree d_v getting multiple merges is $O(\frac{d_v^2}{f^2})$. We readily utilize this result to characterize the probability of K receiving multiple merges as $O(\frac{(d_i+d_j)^2}{f^2})$. □

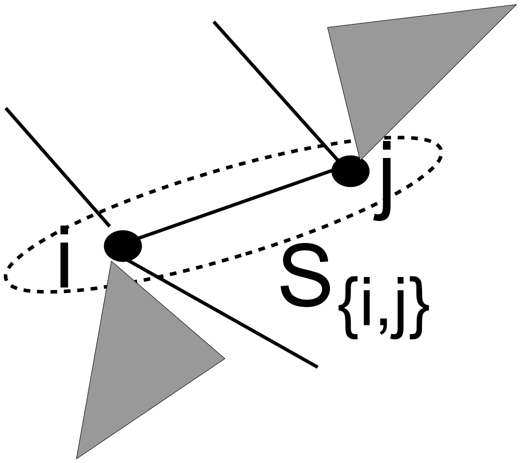

Figure 2. Supernode of facet of dimension 2.

Lemma 3.3 *The probability of facet subsumption is less than the probability of the corresponding supernode getting multiple merges.*

Proof: As an example, let us consider the case when the facet under consideration is an edge (i, j), which means two distinct nodes s and t of the m nodes of the newcomer facet F_f to be merged into the existing network are specifically merged to i and j. This can happen as ($s \rightarrow i$ and $t \rightarrow j$) or ($s \rightarrow j$ and $t \rightarrow i$). Now define the supernode K comprised of edge (i, j). Supernode K getting multiple merges can occur in four combinations as shown in Figures 5 and 6: ($s \rightarrow i$ and $t \rightarrow j$) or ($s \rightarrow j$ and $t \rightarrow i$), and both nodes selecting to merge to the same nodes ($s \rightarrow i$ and $t \rightarrow i$) or ($s \rightarrow j$ and $t \rightarrow j$). Hence, the set of events corresponding to supernodes getting multiple merges is a superset of the set of events corresponding to actual subsumption of the corresponding facets.

Next, consider (an existing) facet $F^{(L)}$ of dimension L, with $m \geq L$ nodes of the newcomer facet F_f being merged to the existing SC. Define the supernode corresponding to $F^{(L)}$ as K_L. Now, L distinct nodes of F_f can merge to the supernode K_L in L^L distinct combinations which lead to a multiple edge merge to K_L. On the other hand, only $L!$ of these combinations, i.e., a permutation of the L distinct nodes of $F^{(L)}$ result in an actual subsumption. □

Lemma 3.4 *The likelihood of subsumption of a facet reduces as facet size increases.*

Proof: Note that for any facet with dimension L, the equivalent facet degree of the corresponding supernode F_S is given by:

$$d_S = \sum_{i=1}^{L} d_i, \tag{9}$$

which naturally implies that the equivalent facet degree of a supernode increases with the dimension of the originating facet. However, the number of multiple merges that is required for the subsumption for a facet of dimension L is greater than or equal to L. Accordingly, this probability is $O(\frac{d_S^L}{f^L})$. Even though the supernode facet degree increases with L, which suggests a higher likelihood of multiple merges, the multiple merges which can subsume a facet decreases with facet size.

Note that on average, the equivalent facet degree d_S increases linearly with facet dimension L. Let us assume that $d_S \approx \kappa L$, where κ is a constant. Then, the likelihood of multiple merges to a supernode corresponding to a facet of dimension L can be bounded as $O(\frac{\kappa^L L^L}{f^L})$.

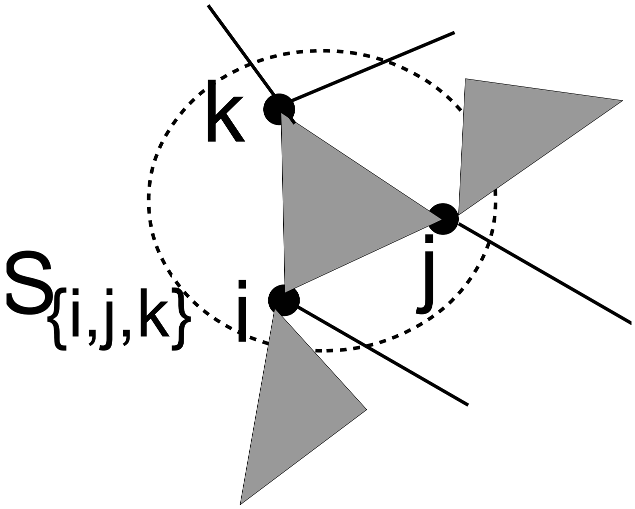

Figure 3. Supernode of facet of dimension 3.

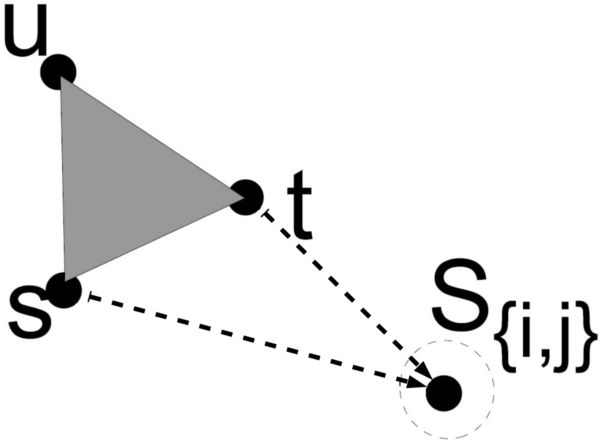

Figure 4. A supernode getting multiple merges. Here the newcomer facet (s,t,u) subsumes edge (i,j) in the existing SC.

On the other hand, recall from Lemma 3.3 that for a facet of dimension L, only a $\frac{L!}{L^L}$ fraction of the supernode multiple merges correspond to an actual subsumption event. Accordingly, the overall likelihood of subsumption can be approximated as:

$$\frac{\kappa^L L! L^L}{L^L f^L}, \tag{10}$$

which is maximized for $L = 2$ (edge). This approximation is based on the assumption that all multiple edge merge events are of equal probability. In practice, while this is likely not the case, still $O(\frac{\kappa^L L^L}{f^L})$ decreases with increasing L, since $\frac{\kappa L}{f} < 1$. □

Lemma 3.5 *The probability of more than one facet being subsumed simultaneously is bounded from above by* $O(\frac{(d_i+d_j+d_k)^3}{f^3})$*, for* $(i,j,k) = \arg\max_{i,j,k \in V}(d_i + d_j + d_k)$.

Proof: The minimum number of merges to a supernode which results in more than one facet subsumptions is equal to 3. This occurs when three edges connect with each other to form an empty triangle. Note that two edges sharing a

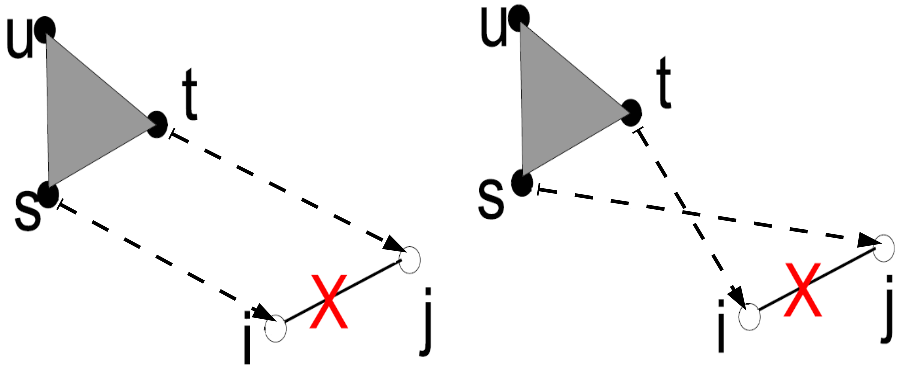

Figure 5. Multiple supernode merges resulting in subsumption

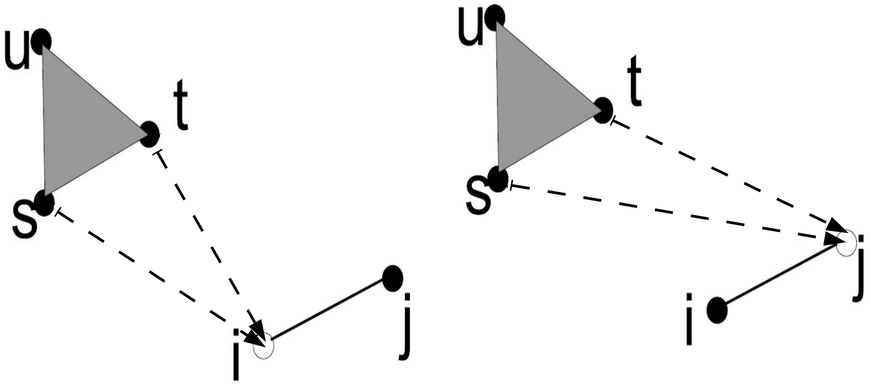

Figure 6. Multiple supernode virtual connections/merging selections not resulting in subsumption

common node also necessitates 3 merges by the incoming facet, but its supernode would have a lower facet degree. Since the likelihood of getting 3 merges is $O(\frac{d_S^3}{f^3})$, this quantity becomes vanishingly small as the SC grows. We also note that it is less likely that an incoming facet will get more than 3 merges to a supernode. □

Lemma 3.6 *The probability of L facets being subsumed simultaneously is upper bounded by the probability of a dimension-L facet being subsumed.*

Proof: Consider L facets of dimension $L-1$ connecting such that they would form a facet of dimension L except that there is a *hole*; e.g. three edges connected to form an empty triangle, or four filled triangles resulting in an empty tetrahedron. The probability of subsumption of such combined structures of L facets can be analyzed by using the supernode technique, and can be shown to be negligible for large SCs. □

Remark 3.1 *The supernode method can be also used for analyzing the probability of subsumption of incoming facets by the existing simplicial complex.*

4 Pseudocodes for GeneSCs

Algorithm GENESCS $(z(\cdot), c, \beta, |V|)$

$f \leftarrow 0$ ▷ Initialize facet counter
while $|V(f)| \leq |V|$ **do**
  $s \leftarrow$ RANDOMSAMPLE$(z(\cdot))$ ▷ Randomly generate size of new facet from distribution $z(s)$
  $F_f \leftarrow$ FACET(s) ▷ Generate $(f+1)$-th facet, with size s
  $nv = \lceil (\frac{|F(f)|+1}{c})^{\frac{1}{\beta}} \rceil$ ▷ Target number of nodes after $(f+1)$-th step following growth equation $|F| = c|V|^{\beta}$.
  $newv = nv - |V(f)|$ ▷ Number of new nodes introduced, i.e., not to be connected to S
  $mergev = s - newv$ ▷ Number of nodes in F_f that need to be merged with S
  while $mergev > 0$ **do**
    $u =$ SELECTNODE(f) ▷ Pick next node in F_f
    $v =$ RANDOMNODE$(S, \frac{f_d(i)}{\sum_{i \in V(f)} f_d(i)})$ ▷ Preferential attachment on facet degree distribution of S
    Merge nodes u and v▷ To prevent a given node in existing SC being selected to merge with multiple nodes of the newcomer facet F_f, we can sample without replacement, or just re-sample the existing SC until we find a node that has not been picked for merging in this step. Note that the probability of this happening vanishes as f grows. (see Appendix 3)
    $mergev \leftarrow mergev - 1$
  end while
  $V(f+1) \leftarrow V(f) \cup$ NODES(F_f)
  ▷ The set of facets after checking if F_f subsumes or is subsumed by one or more existing facets in S
  $F(f+1) \leftarrow$ FACETSUBSUMPTION(S, F_f)
  $S \leftarrow (V(f+1), F(f+1))$
  $f \leftarrow f+1$ ▷ Increment facet counter
end while
return $S = (V(f), F(f))$

Algorithm FACETSUBSUMPTION(S, F_f) ▷ S is represented as a sparse matrix of $|F(f)|$ rows

$S \leftarrow S \cup F_f$ ▷ Add a row to S for now
for $j = 1$ to $|F(f)|$ **do**
  $F_j \leftarrow S(j)$ ▷ j-th row of S
  if $|F_f| \geq |F_j|$ **then** ▷ F_j and F_f are represented as sorted strings of node IDs.
    if SUBSEQUENCE(F_j, F_f) **then** ▷ SUBSEQUENCE(x, y) returns **true** if x is a sub-string of y.
      Add F_j to the delete list D ▷ F_f subsumes F_j
    end if
  else
    if SUBSEQUENCE(F_f, F_j) **then**
      Add F_f to the delete list D ▷ F_f is subsumed by F_j
      break
    end if
  end if
end for
$S \leftarrow S \setminus D$ ▷ Delete all subsumed facets by removing corresponding rows from S; update counters

Figure 7. GENESCS algorithm for generating a random Simplicial Complex ; and Algorithm FACETSUBSUMPTION for computing subsumptions of facets by / into a Simplicial Complex in the pseudocode at the below

References

1. Dorogovtsev, S. N., Mendes, J. F. F. & Samukhin, A. N. Structure of Growing Networks with Preferential Linking. *Physical Review Letters* **85**, 4633–4636 (2000). cond-mat/0004434.
